# Antiatopic dermatitis activity of *Lissachatina fulica* mucin: Network pharmacology analysis and experimental validation

**DOI:** 10.55730/1300-0152.2970

**Published:** 2026-03-16

**Authors:** Atin SUPIYANI, Wasmen MANALU, Lina Noviyanti SUTARDI, Mawar SUBANGKIT, Andriyanto ANDRIYANTO

**Affiliations:** 1 Doctoral Program of Veterinary Biomedical Sciences, School of Veterinary Medicine and Biomedical Sciences, IPB University, Bogor, Indonesia; 2 Division of Physiology, School of Veterinary Medicine and Biomedical Sciences, IPB University, Bogor, Indonesia; 3 Division of Veterinary Pharmacy, School of Veterinary Medicine and Biomedical Sciences, IPB University, Bogor, Indonesia; 4 Division of Pathology, School of Veterinary Medicine and Biomedical Sciences, IPB University, Bogor, Indonesia; 5 Division of Pharmacology and Toxicology, School of Veterinary Medicine and Biomedical Sciences, IPB University, Bogor, Indonesia

**Keywords:** Eplerenone, interleukin-1β, *Lissachatina fulica* mucin, macamide, N-benzyloleamide

## Abstract

**Background/aim:**

*Lissachatina fulica* mucin (LFM) has been used as a nutraceutical, therapeutic agent, and cosmetic ingredient. The bioactivity of mucin depends on bioactive compounds whose composition is strongly influenced by the snail’s diet. This study investigated the bioactive compounds in *L. fulica* mucin as potential therapeutic agents for atopic dermatitis (AD).

**Materials and methods:**

LFM was collected from snails fed long beans and melon rinds for 4 weeks, freeze-dried, and analyzed using liquid chromatography–mass spectrometry followed by network pharmacology (NP) analysis to identify its bioactive compounds. A total of 60 BALB/c mice were sensitized on the dorsal skin with 1% 2,4-dinitrochlorobenzene and subsequently treated with dexamethasone or LFM cream (5% or 10%), which was applied daily for 7 days. Skin samples were collected on days 0, 3, 5, and 7 for immunohistological analysis.

**Results:**

The results demonstrated that LFM contained 61 bioactive compounds, 16 of which interacted with seven target proteins: CCL2, TNF, PTGS2, SYK, MMP9, AKT1, and ICAM1. The NP analysis identified three major bioactive compounds in LFM—macamide, N-benzyloleamide, and a compound tentatively annotated as eplerenone—that potentially targeted CCL2 and TNF, which may serve as key regulators associated with the anti-AD effects of LFM. LFM 5% cream significantly reduced Atopic Dermatitis Severity Index (ADSI) scores and interleukin-1β (IL-1β) expression on day 5 (p < 0.05). LFM normalized epidermal thickness and increased the numbers of macrophages and mast cells (p < 0.05), suggesting a potential role in tissue remodeling and inflammation resolution during skin recovery.

**Conclusion:**

LFM demonstrated potential anti-AD effects by modulating CCL2- and TNF-associated pathways. LFM 5% cream reduced ADSI scores and IL-1β expression while normalizing epidermal thickness, potentially through immunomodulatory responses during the skin recovery phase.

## 1. Introduction

Atopic dermatitis (AD) is a chronic inflammatory skin disorder that affects both children and adults and remains challenging to treat. Acute manifestations include pruritus, erythema, edema, vesiculation, and ulceration. In Indonesia, AD ranks fifth among pediatric skin diseases, with a prevalence of 10%–20% in children and 1%–3% in adults (Izdihar et al., 2024). Prevention focuses on enhancing the skin barrier, addressing immune dysregulation, and controlling allergen exposure (Chu et al., 2023). Current treatments include topical therapies such as glucocorticoids, calcineurin inhibitors, antihistamines, phototherapy, antimicrobials, antiseptics, and emollients (Samandi and Bird, 2021). However, antihistamines have shown limited efficacy in relieving pruritus (Cai et al., 2022), whereas glucocorticoids, which remain the primary treatment option, are not recommended for long-term use because of their adverse effects (Ronchetti et al., 2021).

Substantial research has investigated the therapeutic potential of both plant- and animal-derived natural products for the treatment of AD. Mucin, a naturally occurring animal-derived product, is secreted by mucus-producing cells in response to physiological stimuli. It is a highly O-glycosylated molecule with gel-like properties (Johansson and Hansson, 2016). *Lissachatina fulica*, commonly known as the giant African land snail, produces mucin that has been used for medicinal and cosmetic purposes, particularly in wound healing and antiaging applications. *Lissachatina fulica* is an invasive detritivorous species with considerable dietary flexibility and is capable of consuming a wide variety of leaves and fruits, thereby contributing to plant damage (Pratiwi et al., 2022). Previous studies have shown that snails fed organic vegetable and fruit waste exhibit greater body mass and shell growth than wild snails (Supiyani et al., 2026). However, limited information is available regarding the composition of bioactive compounds in the mucus of *Lissachatina fulica* fed organic waste-based diets.

Bioactive compounds derived from snail mucin have been reported to promote wound healing and enhance skin cell proliferation (Singh et al., 2024). The precise mechanisms underlying the antiinflammatory effects of *Lissachatina fulica* mucin remain unclear. Further studies are needed to clarify its biological roles and standardize its therapeutic activity for potential use against AD. This study investigated the in vivo effects and potential mechanisms of action of bioactive compounds derived from *Lissachatina fulica* mucin (LFM) as candidate therapeutic agents for AD. A quantitative approach was used to assess inflammatory cell infiltration and interleukin-1β (IL-1β) expression in the dermis.

## 2. Materials and methods

### 2.1. Animal treatment

All research animals were maintained at the Laboratory Animal Management Unit, School of Veterinary Medicine and Biomedical Sciences, IPB University, under Animal Ethics approval no. 211/KEH/SKE/IV/2024. *Lissachatina fulica* (Bowdich, 1822) was identified and taxonomically verified by the Zoology Laboratory, National Research and Innovation Agency (BRIN), under document no. FR-ZO.09-01/2024. Snails (5–6 cm in length and weighing 20–24 g) were fed melon rind and long bean waste ad libitum. The snails were housed in cages containing planting medium and covered with wire mesh. The cages were maintained at 24–26 °C and 45%–65% relative humidity. After 4 weeks, the snails were fasted for 12 h before mucus collection.

A total of 60 healthy male BALB/c mice aged 2 months were used for the in vivo experiments. The mice were housed in cages measuring 30 × 20 × 15 cm with wooden bedding and were fed a commercial pellet diet. During the acclimatization period, the mice received ivermectin (0.1 mg/kg body weight) as an anthelmintic treatment. Atopic dermatitis-like skin lesions were induced by the topical application of 1% 2,4-dinitrochlorobenzene (DNCB) solution to the shaved dorsal skin for 7 consecutive days, followed by coverage with sterile gauze and adhesive plaster (Jang et al., 2020). The mice were divided into five treatment groups: DNCB, DNCB + dexamethasone, DNCB + cream base, DNCB + 5% LFM cream, and DNCB + 10% LFM cream. The assigned treatments were applied daily to the dorsal skin for 7 days. Blood and skin tissue samples were collected on days 0, 3, 5, and 7 for analysis.

### 2.2. LFM extraction and bioactive compound identification

Snail mucus was collected by gently rubbing the pedal muscle with a glass spatula. The collected mucus was transferred into sterile sample tubes, and crude *L.fulica* mucin (LFM) was obtained by freeze-drying at −53 °C for 72 h at the Genomics Laboratory, National Research and Innovation Agency, Cibinong, Indonesia.

The bioactive compounds of LFM were analyzed using an UHPLC Vanquish Tandem Q Exactive Plus Orbitrap HRMS system (Thermo Fisher Scientific, Waltham, MA, USA) coupled with liquid chromatography–mass spectrometry (LC-MS) analysis. A 2.5 mg aliquot of LFM extract was dissolved in 0.5 mL of methanol (MeOH) and filtered through a 0.2 μm nylon membrane. Chromatographic separation was performed using an Accucore Phenyl Hexyl column (100 mm × 2.1 mm, 1.5 μm; Thermo Fisher Scientific, Waltham, MA, USA). The column and sample tray temperatures were maintained at room temperature and 30 °C, respectively. The mobile phases consisted of H_2_O containing 0.1% formic acid (A) and acetonitrile containing 0.1% formic acid (B), which were applied using a gradient elution program. The gradient program was as follows: 0–1 min, 5% B; 1–25 min, 5%–95% B; 25–28 min, 95% B; and 28–33 min, 5% B. Additional operating conditions included a spray voltage of 3.8 kV, a capillary temperature of approximately 320 °C, sheath gas and auxiliary gas flow rates of 15 and 3 mL/min, respectively, and full MS/dd-MS^2^ acquisition in positive ionization mode. Data processing was performed using the ChemSpider database (Royal Society of Chemistry, Cambridge, UK) and the mzCloud spectral database (Thermo Fisher Scientific, Waltham, MA, USA). Predominant compounds were identified based on high-confidence spectral matching scores (>80%) and relative peak-area abundance. Although LC-MS peak intensity does not necessarily correlate linearly with absolute compound concentration, these criteria were used to prioritize biologically relevant candidates for subsequent network pharmacology analysis.

### 2.3. Network pharmacology analysis of bioactive compounds identified in LFM

The chemical profiles and structural information of the bioactive compounds identified in LFM were verified and cross-referenced using the PubChem database (Kim et al., 2023). Target protein prediction for compounds identified in LFM was performed using SwissTargetPrediction (Daina et al., 2019) with a probability threshold of >0.05. STRING (Szklarczyk et al., 2023) was used to construct a protein–protein interaction (PPI) network involving LFM-derived targets and AD-related proteins. The predicted target proteins were uploaded to STRING to generate PPI data using *Homo sapiens* as the reference organism and a high-confidence interaction score threshold of 0.700. Cytoscape version 3.10.4 (Cytoscape Consortium, San Diego, CA, USA) was used to visualize compounds and target proteins as nodes and their interactions as edges. To identify hub genes, network nodes were analyzed based on their topological properties, and nodes with a degree value of <10 were excluded from further analysis. CCL2 and TNF were prioritized for further discussion based on their high degree centrality and their well-established involvement in AD-associated inflammatory signaling pathways.

### 2.4. Preparation of LFM cream

The cream base formulation consisted of cetyl alcohol, triethanolamine, stearic acid, propylene glycol, paraffin, and methylparaben, which were thoroughly dispersed in distilled water. LFM extract was incorporated into the cream base at concentrations of 5% and 10% (w/w).

### 2.5. Atopic Dermatitis Symptom Index

The AD symptom score was determined according to the Atopic Dermatitis Severity Index (ADSI), which is based on the sum of the scores for pruritus, erythema, excoriation, exudation, and lichenification. ADSI scores were classified as follows: 0 to <2, clear or almost clear; 2 to <6, mild; 6 to <9, moderate; and 9 to 15, severe (Silverberg et al., 2020).

### 2.6. Quantification of inflammatory cells

Skin tissue sections were stained with hematoxylin and eosin and examined under a light microscope to evaluate inflammatory cell infiltration. ImageJ software (National Institutes of Health, Bethesda, MD, USA) was used to quantify cells in five randomly selected fields at 100× magnification.

### 2.7. Analysis of IL-1β expression

Skin tissues sections were immunohistochemically stained using an antiinterleukin-1β rabbit polyclonal antibody (CAB20527; Assay Genie, Dublin, Ireland). IL-1β expression was evaluated and quantified in five randomly selected fields at 100× magnification using ImageJ software (National Institutes of Health, Bethesda, MD, USA).

### 2.8. Data analysis

All data were analyzed using GraphPad Prism version 10 (GraphPad Software, Boston, MA, USA). Data were analyzed using a two-way analysis of variance (ANOVA), followed by Tukey’s multiple-comparison test when significant differences were detected. Statistical significance was set at p < 0.05.

## 3. Results

### 3.1. Bioactive compounds identified in LFM

The chemical composition of hydrolyzed LFM was characterized using liquid chromatography–mass spectrometry (LC–MS). The predominant compounds were identified based on their relatively high integrated peak areas compared with those of other detected constituents in the LFM extract (Table 1).

As shown in Table 1, out of the 61 compounds identified, five predominant compounds were prioritized based on their relative peak areas, reflecting their relative abundance within the extract: L-(+)-valine (C_5_H_11_NO_2_), (9Z)-9-octadecenamide (C_18_H_35_NO), erucamide (C_22_H_43_NO), palmitamide (C_16_H_33_NO), and safingol (C_18_H_39_NO_2_). L-(+)-valine was identified as the most abundant compound in the mucin extract. Valine is a glucogenic amino acid that plays important roles in energy metabolism and protein synthesis (Stewart et al., 2020). Valine has been reported to contribute to protein synthesis and tissue repair processes under specific physiological conditions. (9Z)-9-octadecenamide has been investigated for its antioxidant and hypolipidemic properties (Hossain et al., 2024). Erucamide, a primary fatty amide, has been reported to exhibit antibacterial properties (Bao et al., 2025). Palmitamide, a long-chain fatty acid amide, has been reported to exhibit antiinflammatory and immunomodulatory effects (Sá and Castor, 2023). Safingol, a saturated derivative of sphingosine, has been implicated in the regulation of cell migration, proliferation, inflammation, and cell death (Companioni et al., 2021). Further analyses are needed to clarify the potential roles of these compounds in AD-related biological processes using network pharmacology approaches.

### 3.2. Network pharmacology analysis of bioactive compounds identified in LFM

**Figure 1:**
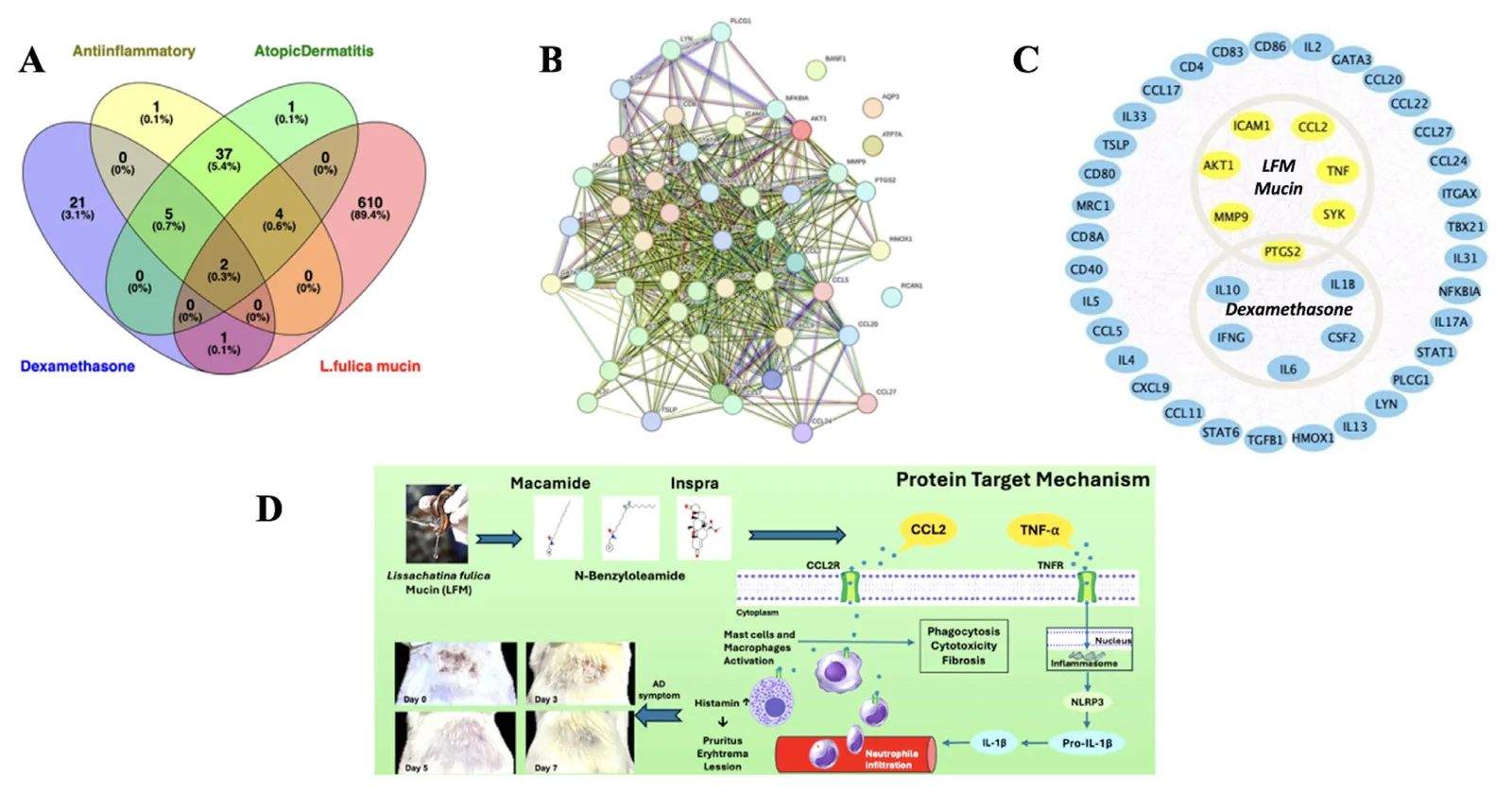
Network pharmacology analysis of *Lissachatina fulica* mucin (LFM). (A) Venn diagram showing 49 overlapping target proteins. (B) Protein–protein interaction (PPI) network. (C) LFM-associated target proteins related to inflammatory regulation and atopic dermatitis (yellow nodes). (D) Predicted associations of macamide, N-benzyloleamide, and eplerenone with CCL2- and TNF-related pathways.

A comprehensive analysis of 61 bioactive compounds identified in LFM revealed 617 predicted target proteins (Figure 1A). A total of six target proteins associated with inflammatory regulation were identified: AKT1, ICAM1, MMP9, SYK, CCL2, and TNF. Among the identified targets, four proteins were associated with AD: AKT1, ICAM1, MMP9, and SYK. PTGS2 was identified as a shared target between LFM-derived compounds and dexamethasone. The Venn diagram illustrates the overlap between two common targets, CCL2 and TNF, identified in the LFM, AD-related, and dexamethasone-associated target datasets (Figure 1A).

The network pharmacology analysis indicated that 16 of the 61 bioactive compounds identified in LFM were associated with seven target proteins implicated in inflammatory regulation and AD pathogenesis (Figure 1). These targets overlapped with those associated with dexamethasone, a therapeutic agent commonly used in the management of AD. Additional details are provided in Table 2.

The findings suggest that bioactive compounds identified in LFM may influence biological pathways involving CCL2 and TNF. Li et al. (2022) reported that CCL2 and TNF are key proinflammatory mediators associated with M1-like macrophage polarization through the NF-κB/NLRP3 signaling pathway. TNF-α, IL-1β, and monocyte chemoattractant protein-1 (MCP-1) are proinflammatory mediators that play important roles in host defense and inflammatory responses to infection and tissue injury (Kumboyono et al., 2022). Therefore, subsequent in vivo experiments were conducted to evaluate the effects of LFM on inflammatory cell infiltration and IL-1β expression in mice with DNCB-induced AD-like skin lesions.

Although the in vivo validation primarily focused on IL-1β expression, this cytokine is closely associated with the CCL2- and TNF-related signaling pathways identified through NP analysis. In the pathogenesis of AD, IL-1β is recognized as an important upstream proimflammatory cytokine that can activate NF-κB signaling, thereby promoting the expression of TNF-α and the chemokine CCL2 (MCP-1). Although CCL2 is commonly associated with inflammatory cell recruitment, its involvement in tissue repair and the transition from inflammation to resolution has also been reported under specific physiological conditions. The significant increase in macrophage numbers observed in the LFM-treated groups, particularly in the 5% LFM group, suggests that the CCL2-related pathway identified through NP analysis may be associated with macrophage recruitment to the site of injury. Macrophages have been reported to contribute to the resolution of inflammation through the clearance of cellular debris and the production of tissue repair-associated mediators. Taken together, the reduction in IL-1β expression and the increased macrophage infiltration observed following LFM treatment suggest that LFM may contribute to the modulation of inflammatory responses and the promotion of skin tissue recovery.

### 3.3. Clinical signs and ADSI scores

**Figure 2:**
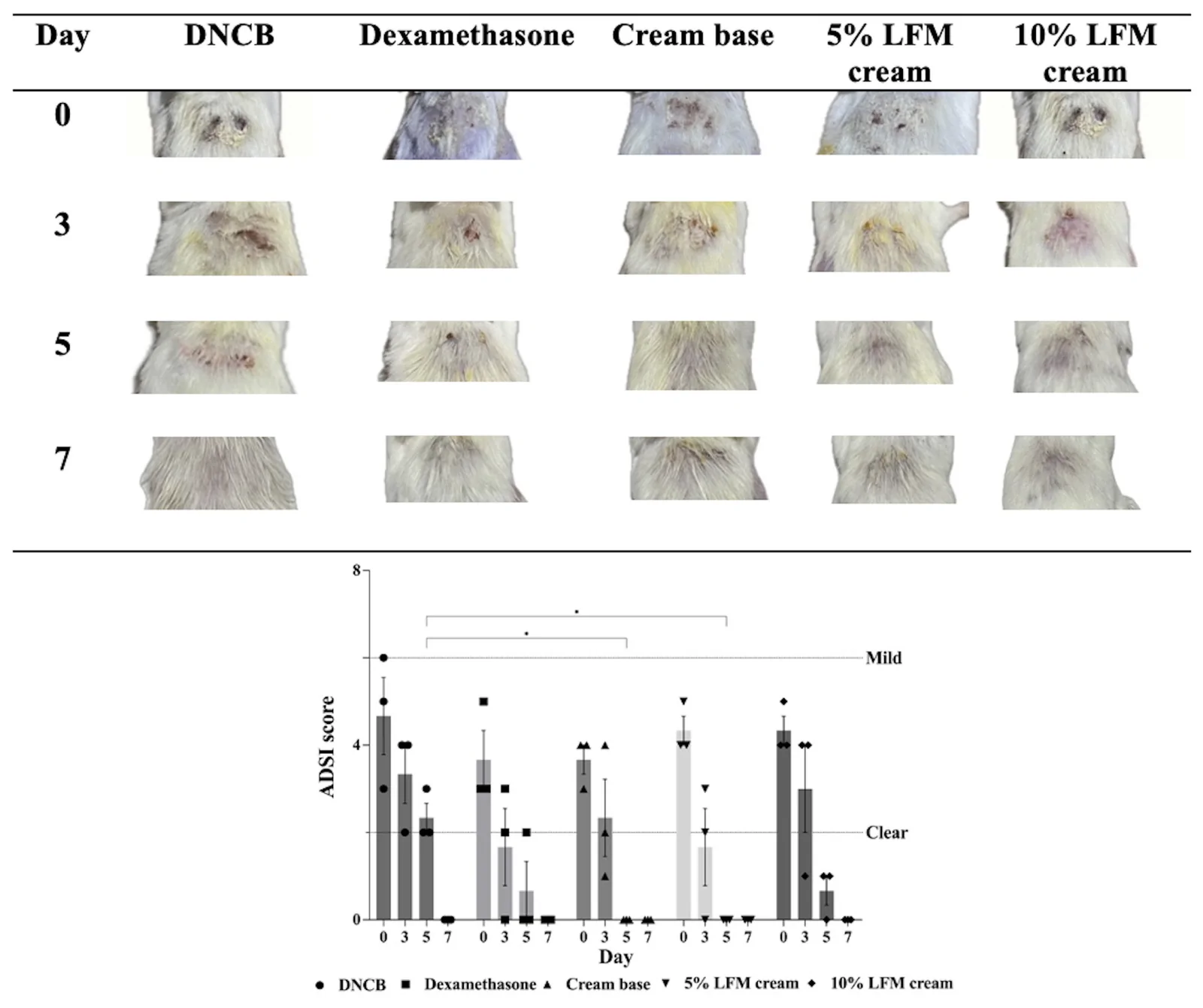
Representative clinical signs and ADSI scores of AD experimental mice treated with *Lissachatina fulica* mucin (LFM) cream. *Significant differences were determined using Tukey’s multiple-comparison test (p < 0.05).

Mice with DNCB-induced AD-like skin lesions exhibited the principal clinical manifestations of pruritus, erythema, excoriation, and skin thickening. Topical application of 1% DNCB successfully induced AD-like skin lesions in vivo (Figure 2).

The progression of AD-like skin lesions was visually documented from day 0 to day 7 (Figure 2). On day 0, all sensitized groups exhibited mild AD-like lesions characterized by marked erythema, hemorrhagic lesions, and dry crust formation. By day 3, erythema had decreased, whereas skin thickening and excoriation became more prominent. According to the ADSI classification, all mice were categorized as having mild AD-like lesions (ADSI 2 to <6) on days 0 and 3 (Figure 2). On day 5, the DNCB group remained within the mild category, whereas the remaining treatment groups were classified as clear or almost clear (ADSI <2). No visible AD-like lesions were observed on day 7. Topical DNCB exposure induced skin irritation characterized by pruritus, erythema, excoriation, and lichenification (Figure 2). The DNCB-induced AD-like model yielded ADSI scores ranging from 2 to <6, corresponding to the mild disease category. The inflammatory response, which is characterized by the release of inflammatory mediators, represents a fundamental biological reaction to infection or tissue injury. This process serves to eliminate cellular debris, contain pathogenic organisms, and limit infection or tissue damage. Clinical manifestations of inflammation include localized redness, swelling, pain, and increased temperature at the affected site (da Silva et al., 2022).

### 3.4. Epidermal and dermal thickness

**Figure 3:**
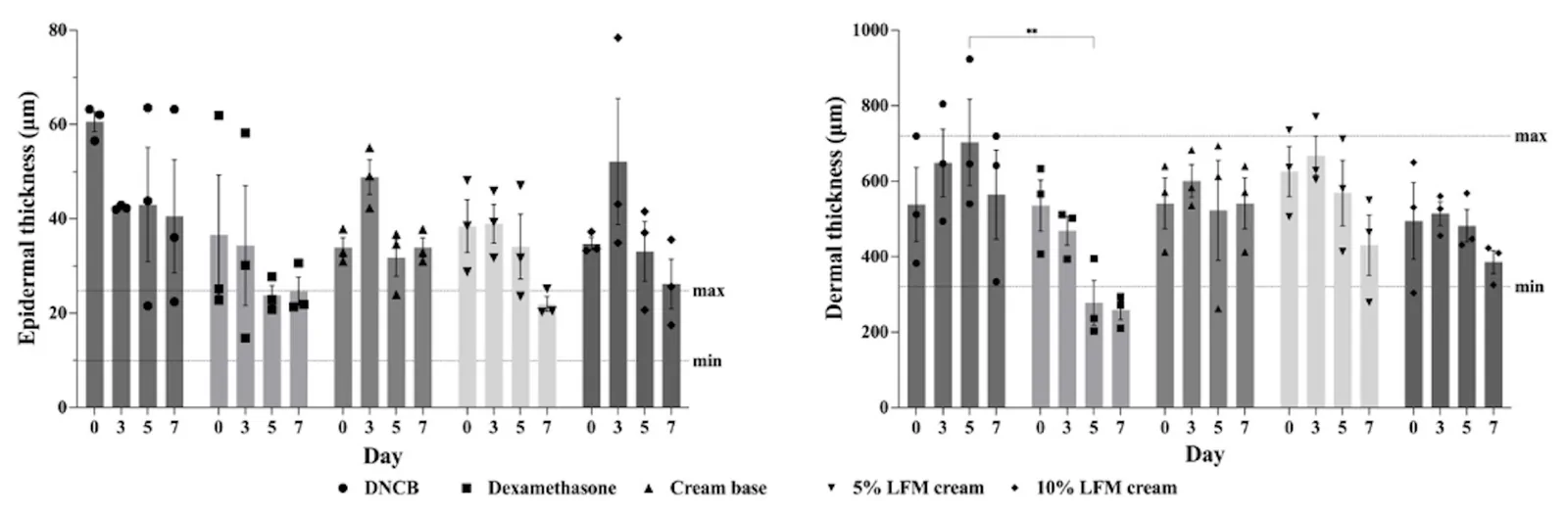
Epidermal and dermal thickness in AD experimental mice treated with *Lissachatina fulica* mucin (LFM) cream. *Significant differences were determined using Tukey’s multiple-comparison test (p < 0.05).

DNCB-induced AD-like skin lesions and the associated inflammatory cell response can lead to histological alterations in skin tissue. These alterations can be observed through changes in the thickness of the epidermal and dermal layers, as shown in Figure 3.

AD-associated skin inflammation is characterized by epidermal hyperplasia (acanthosis), dermal thickening, inflammatory cell infiltration, parakeratosis, and spongiosis (Alam et al., 2023). Histological changes in skin tissue, particularly the thickness of the epidermal and dermal layers, were analyzed to evaluate the structural effects of LFM (Figure 3). No significant differences in epidermal thickness were observed among the experimental groups throughout the 7-day study period (p > 0.05). Despite the absence of statistically significant differences, epidermal thickness in the 5% and 10% LFM groups remained within the baseline physiological range following DNCB induction.

In contrast, significant variations in dermal thickness were observed (Figure 3, right). The DNCB-only group exhibited the greatest dermal thickness on day 3, consistent with inflammatory edema. By day 7, the dexamethasone-treated group exhibited a significant reduction in dermal thickness (p < 0.01), approaching the lower limit of the physiological range. This finding may reflect early dermal thinning, which has been reported following prolonged corticosteroid exposure. Notably, dermal thickness in the 5% and 10% LFM groups remained within the physiological range throughout the treatment period. These findings suggest that LFM does not markedly alter epidermal structure and may contribute to the maintenance of dermal architecture during the recovery period.

### 3.5. Inflammatory cell counts

**Figure 4:**
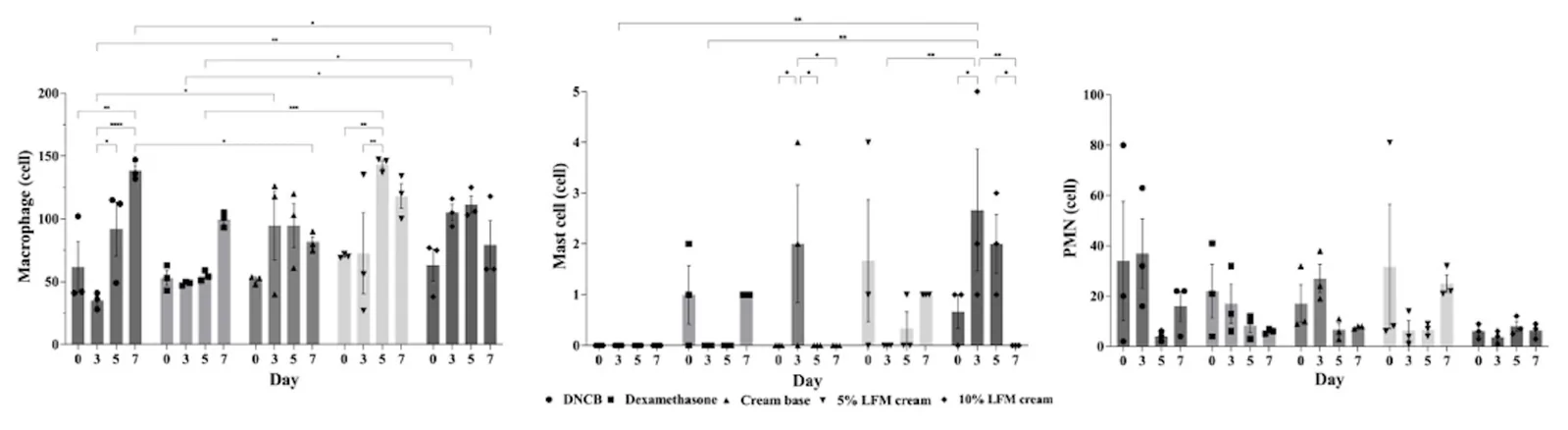
Numbers of macrophages, mast cells, and PMNs in the skin tissue of AD experimental mice treated with *Lissachatina fulica* mucin (LFM) cream. *Significant differences were determined using Tukey’s multiple-comparison test (p < 0.05).

The inflammatory response associated with AD involves the activation and recruitment of mast cells, polymorphonuclear leukocytes (PMNs), and macrophages within the skin. Figure 4 shows a statistically significant increase in mast cell and macrophage numbers in the 5% and 10% LFM cream groups on days 3 and 5 (p < 0.05).

LFM treatment was associated with significant changes in macrophage and mast cell number (p < 0.05). Macrophage numbers were significantly increased on days 3, 5, and 7 (p < 0.05). On day 5, the 5% LFM cream group exhibited significantly higher macrophage numbers than the other treatment groups (p < 0.05). A statistically significant increase in mast cell numbers was observed in the 10% LFM cream group on day 3 (p < 0.05). Mast cells and macrophages play important roles in regulating inflammatory responses and tissue homeostasis. The increased mast cell numbers observed in the 5% and 10% LFM cream groups on day 3 suggest that LFM may influence mast cell recruitment or retention during the early inflammatory response. Activated mast cells can release histamine, which contributes to pruritus and scratching behavior. Histamine release promotes vasodilation and increases vascular permeability, facilitating leukocyte recruitment to inflamed tissues and contributing to erythema. Skin irritation can contribute to lesion formation. Persistent skin injury may contribute to hyperpigmentation, hyperkeratosis, and lichenification.

Increased macrophage numbers were observed in the 5% and 10% LFM cream groups on day 5 (p < 0.05). During inflammation, macrophages release cytokines and chemokines that contribute to the regulation of inflammatory responses (Wiratmini et al., 2025). The increased macrophage numbers observed in the 5% and 10% LFM groups on day 3 suggest that LFM may modulate cellular responses involved in tissue repair and inflammatory regulation. The concurrent increase in macrophage and mast cell numbers observed during LFM treatment may reflect dynamic changes in the local immune response during tissue recovery. It may be hypothesized that LFM influences macrophage functional phenotypes; however, macrophage polarization was not directly evaluated in the present study. Furthermore, the limited effect of LFM on PMN counts suggest that its biological effects may not primarily involve the direct suppression of acute neutrophilic infiltration.

### 3.6. IL-1β expression

**Figure 5:**
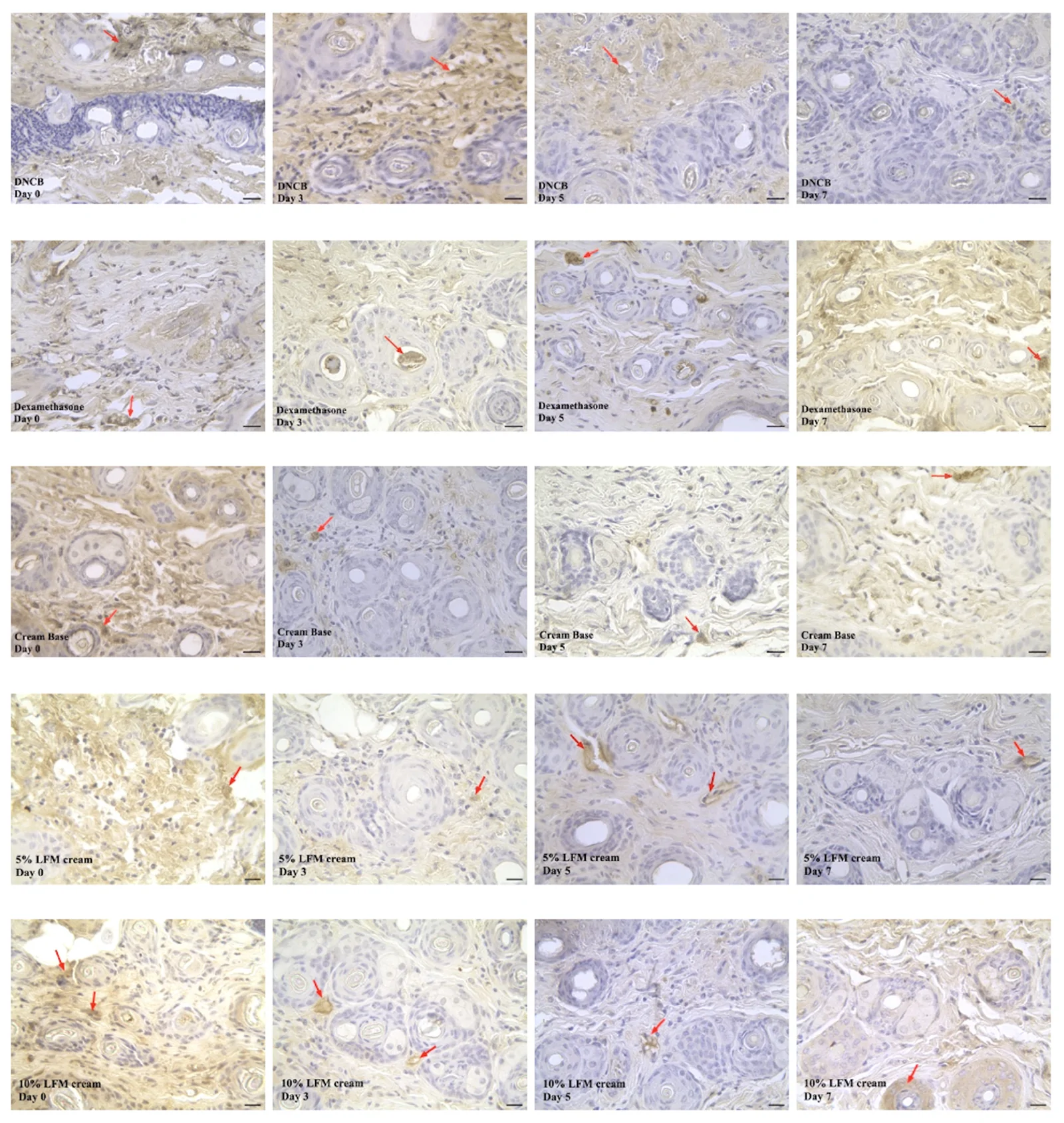
Representative photomicrographs of IL-1β immunohistochemical expression in the skin tissue of AD experimental mice treated with *Lissachatina fulica* mucin (LFM) cream. Arrows indicate IL-1β-positive staining. Scale bar = 5 μm; magnification = 400X.

Positive immunohistochemical staining for IL-1β appeared as brown deposits within the skin tissue (Figure 5), with increased staining intensity indicating higher IL-1β expression.

**Figure 6:**
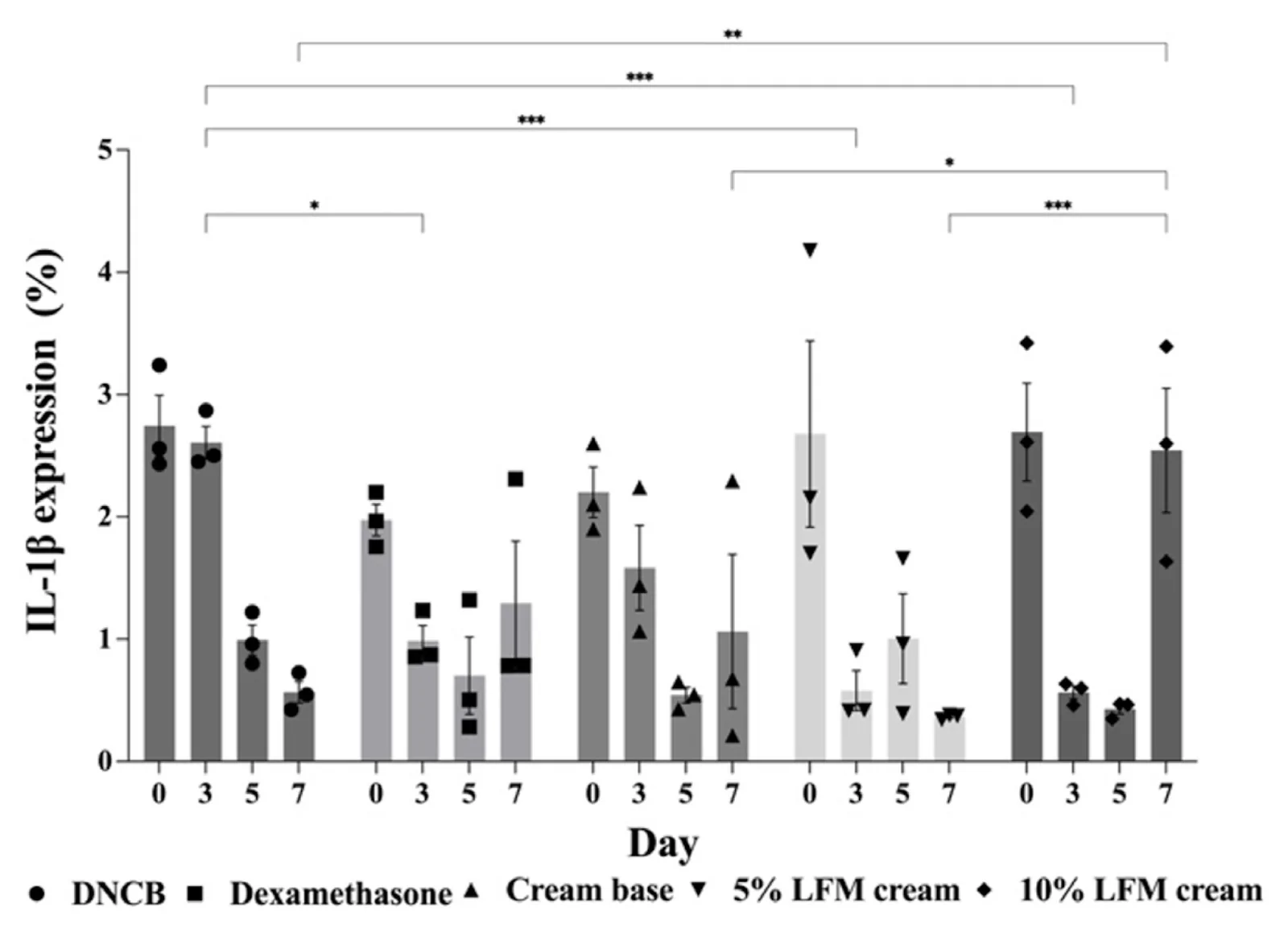
Quantification of IL-1β expression in the skin tissue of AD experimental mice treated with *Lissachatina fulica* mucin (LFM) cream. * Significant differences were determined using Tukey’s multiple-comparison test (p < 0.05).

The inflammatory process in the skin stimulates the production of IL-1β, a key proinflammatory cytokine. As shown in Figure 5, IL-1β expression was initially elevated in all groups and decreased by day 3. However, increased staining intensity was observed in the dexamethasone- and LFM-treated groups on day 7, indicating altered IL-1β expression during the later stages of tissue recovery. Quantification of IL-1β expression was performed to evaluate differences among treatment groups, as shown in Figure 6.

IL-1β expression increased on day 7 in the dexamethasone and 10% LFM cream groups, whereas an increase was observed beginning on day 5 in the 5% LFM cream group (p < 0.05). These findings suggests that biological changes associated with the recovery process may have occurred earlier in the 5% LFM cream group, where a significant increase in IL-1β expression was observed on day 5 (p < 0.05). The 5% LFM cream exhibited a more consistent temporal pattern of IL-1β expression than the 10% formulation. The increased IL-1β expression observed on day 7 in the 10% LFM group may indicate a concentration-dependent biological response. However, the mechanisms underlying this observation were not investigated in the present study and therefore remain speculative.

## 4. Discussion

In atopic dermatitis, mutations in the filaggrin gene (*FLG*) contribute to impaired skin barrier function and increased susceptibility to environmental allergens (Afshari et al., 2024). DNCB is widely used to induce AD-like skin lesions because it causes skin irritation and impairs barrier function. The improved histological outcomes observed in the LFM cream-treated groups may be associated with the biological activities of the bioactive compounds present in mucin.

Network pharmacology analysis identified macamide, N-benzyloleamide, and a compound tentatively annotated as eplerenone as candidate bioactive compounds associated with CCL2- and TNF-related pathways. Macamide has been reported to exhibit diverse biological activities, including antioxidant and immunomodulatory effects (Zha et al., 2021). N-benzyloleamide has been reported to reduce malondialdehyde levels and protect against oxidative stress in experimental models (Ybañez-Julca et al., 2022). Eplerenone has been reported to exert pleiotropic effects, including improvements in vascular endothelial function and modulation of inflammatory responses. These effects may be related to mineralocorticoid receptor antagonism, which has been associated with reductions in aldosterone-mediated vascular dysfunction and inflammation (Mostafa and Salama, 2023).

The duration and outcome of an inflammatory response are influenced by the resolution phase, during which inflammatory processes are actively regulated and terminated. This process is mediated by several mechanisms, including autophagy, which facilitates the clearance of damaged cells and organelles (Fullerton and Gilroy, 2016). The inflammatory resolution process consists of three overlapping phases: proinflammatory, resolution, and repair. In the present study, IL-1β expression in skin tissue was used as an indicator of local inflammatory activity in mice with DNCB-induced AD-like skin lesions. The resolution phase is characterized by the activity of reparative and regulatory macrophages, which secrete cytokines, effector molecules, and growth factors that contribute to tissue repair processes (Dwyer and Turnquist, 2021).

LFM treatment was associated with changes in inflammatory cell numbers and IL-1β expression during the experimental period. These effects may be related to bioactive compounds identified in LFM, including macamide, N-benzyloleamide, 1,4-dimethylcyclohexane, 1-(3-methylpentyl)-4-phenoxybenzene, N,N,2-trimethyl-5H-1,3-benzodiazepin-4-amine, 2-(3-phenylpropyl)pyridine, artocarpin, and capsiamide, which were predicted by network pharmacology analysis to be associated with CCL2- and PTGS2-related pathways. The compound tentatively identified as eplerenone was predicted to be associated with TNF- and PTSG2-related pathways in the network pharmacology analysis. Stachydrine (proline betaine) has been reported to reduce the secretion of IL-1β and TNF-α, thereby exhibiting antiinflammatory properties (He et al., 2024). Palmitamide has been reported to modulate inflammatory processes, including cytokine production, mast cell degranulation, and microglial activation, while reducing oxidative stress (Das and Balakrishnan, 2025). Safingol has been reported to inhibit sphingosine kinase 1 (SphK1), a signaling molecule involved in inflammation and cell survival pathways. Consequently, the significant increase in macrophage and mast cell counts observed during LFM treatment is indicative of an active reparative remodeling phase rather than persistent pathological inflammation; in these late stages of skin recovery, these immune cells transition to play a crucial role in orchestrating tissue repair and accelerating the transition toward complete inflammation resolution.

The findings of the present study suggest that LFM may modulate inflammatory responses and influence macrophage-associated processes involved in tissue homeostasis and repair. These effects may be associated with bioactive compounds identified in LFM, including 2-aminohexadecan-1-ol and N-(2-hydroxyethyl)icosanamide. Evidence from previous studies indicates that 2-aminohexadecan-1-ol enhances nitric oxide production in rat macrophages (Bu et al., 2021), whereas N-(2-hydroxyethyl)icosanamide has been associated with the activation of monocyte chemoattractant protein-1 (MCP-1) signaling (Abdullah et al., 2022). MCP-1 functions as a chemokine that promotes the recruitment of monocytes, memory T cells, and basophils to sites of inflammation (Saksono et al., 2023). The primary goals of AD treatment are to restore and maintain epidermal barrier function while reducing inflammation (Tay et al., 2021). The bioactive compounds identified in LFM may contribute to the maintenance of skin barrier integrity and the modulation of inflammatory responses.

In summary, LFM was associated with improvements in AD-like skin lesions and may exert its effects through pathways involving IL-1β, CCL2, and TNF. At a concentration of 5%, LFM cream reduced ADSI scores and IL-1β expression and was associated with increased macrophage and mast cell numbers during the recovery period. These findings suggest that LFM may exert antiinflammatory effects and contribute to skin tissue recovery following DNCB-induced injury. However, given the potential biological instability of LFM, further studies are required to develop effective preservation strategies and to investigate its long-term effects on skin barrier-associated biomarkers, including filaggrin expression.

## Figures and Tables

**Table 1:** Bioactive compounds identified in LFM by LC-MS analysis.

No	Name	Formula	Mass error (ppm)	Calculated MW (Da)	RT [min]	Relative peak area (%)
1	L-(+)-Valine	C_5_H_11_NO_2_	1.00	117.0791	1.106	1,608,396,150.00
2	(9Z)-9-Octadecenamide	C_18_H_35_NO	−0.45	281.2717	25.492	618,682,301.30
3	Erucamide	C_22_H_43_NO	−1.13	337.3341	30.619	335,544,988.80
4	Palmitamide	C_16_H_33_NO	−0.61	255.2561	28.372	308,106,782.00
5	Safingol	C_18_H_39_NO_2_	−1.10	301.2978	17.376	302,575,334.70
6	4-Aminobenzoic acid	C_7_H_7_NO_2_	0.38	137.0477	1.129	164,366,755.10
7	1-Hexadecanoylpyrrolidine	C_20_H_39_NO	−0.92	309.3029	29.303	157,538,058.80
8	Stearylamine acetate	C_20_H_43_NO_2_	−0.05	329.3294	19.620	153,618,845.00
9	Macamide 1	C_23_H_39_NO	−0.95	345.3028	27.997	143,988,162.70
10	Dodecylamine acetate	C_14_H_31_NO_2_	−0.65	245.2353	12.784	122,044,916.10
11	[2′,7′-bis(3,7-dimethyl octan-3-yl)-9,9′-spirobi[fluorene]-4-yl]carbamic acid	C_46_H_57_NO_2_	1.06	655.4396	24.711	95,308,074.44
12	Guaiacol sulfate	C_7_H_8_O_5_S	3.09	204.0099	31.059	84,723,760.60
13	4-Phosphorosooxy-1,3-thiazole-5-carbonitrile	C_4_HN_2_O_2_PS	−2.58	171.9492	1.301	70,596,071.68
14	Eplerenone	C_24_H_30_O_6_	0.59	414.2045	18.024	62,530,187.96
15	Stearyldiethanolamine	C_22_H_47_NO_2_	0.67	357.3609	21.846	58,585,695.65
16	N-(2-dimethylaminoethyl)-4,6-dimethoxy-2-piridinylmethylthio)-6-phenyl-1,3,5-triazine-2-amino-3-pyridazinylmethylphosphoramidate	C_40_H_57_N_6_O_2_P	1.55	684.4291	27.079	56,808,226.78
17	Artocarpin	C_26_H_28_O_6_	−4.70	436.1865	18.026	55,186,553.13
18	1,4-dimethyl cyclohexane;1-(3-methylpentyl)-4-phenoxy benzene; N,N,2-trimethyl-5H-1,3-benzodiazepine-4-amine	C_38_H_53_N_3_O	2.70	567.4204	29.461	47,781,882.80
19	2a,7-Dihydroxy-3-(hydroxymethyl)-6,6,7b-trimethyl-2,2a,4a,5,6,7,7a,7b-octahydro-1H-cyclobuta[e]inden-2-yl 3-chloro-4,6-dihydroxy-2-methyl benzoate	C_23_H_29_ClO_7_	0.73	452.1605	18.027	42,665,383.89
20	Stachydrine	C_7_H_13_NO_2_	0.70	143.0947	1.147	39,466,412.03
21	Pirbuterol	C_12_H_20_N_2_O_3_	0.61	240.1475	1.537	37,288,012.37
22	2-Aminohexadecan-1-ol	C_16_H_35_NO	0.72	257.2721	17.512	36,694,403.78
23	(11Z,14Z)-eicosadienamide	C_20_H_37_NO	0.23	307.2876	26.553	35,706,282.48
24	N-(2-hydroxyethyl) icosanamide	C_22_H_45_NO_2_	0.83	355.3453	20.216	34,529,031.35
25	1-Stearoyl-2-hydroxy-sn-glycerol-3-PE	C_23_H_48_NO_7_P	1.29	481.3175	22.147	33,876,607.43
26	2-(3-Phenylpropyl) pyridine	C_14_H_15_N	0.34	197.1205	8.628	33,491,851.20
27	Cetaben	C_23_H_39_NO_2_	0.09	361.2981	26.757	32,922,737.71
28	Propane-1-sulfonohydrazide	C_3_H_10_N_2_O_2_S	0.54	138.0464	1.214	32,153,682.75
29	1-Palmitoyl-sn-glycerol-3-phosphocholine	C_24_H_50_NO_7_P	1.45	495.3332	19.91	31,629,568.07
30	1-(6-Nonyl-3-pyridinyl)-1-decanoate	C_24_H_41_NO	0.05	359.3188	28.499	31,177,479.47
31	Butylphosphonic acid	C_29_H_45_O_3_P	0.44	472.3108	29.321	28,166,178.18
32	1-Palmitoyl-2-hydroxy-sn-glycerol-3-PE	C_21_H_44_NO_7_P	1.01	453.2860	19.571	27,971,574.05
33	Tridemorph	C_19_H_39_NO	−0.06	297.3032	30.066	27,702,614.50
34	N-3-Methoxybenzyl9Z,12Z,15Z-octadeca-9,12,15-trienamide	C_26_H_39_NO_2_	0.08	397.2981	26.275	25,465,350.17
35	Myristamide	C_14_H_29_NO	−0.19	227.2249	25.685	24,771,683.24
36	Octylamine	C_8_H_19_N	0.72	129.1518	5.790	23,329,698.28
37	5-guanidino-2-oxopentanoic acid	C_6_H_11_N_3_O_3_	1.37	173.0803	1.157	21,901,489.48
38	Spisulosine	C_18_H_39_NO	0.69	285.3034	19.786	21,680,282.25
39	1-(alpha-D-Glucopyranosyluronosyl)-3-[(1)-1-methyl-5-oxo-2-pyrrolidinyl]pyridinium	C_16_H_20_N_2_O_7_	0.72	352.1273	8.298	20,776,129.95
40	DL-Carnitine	C_7_H_15_NO_3_	0.68	161.1053	1.105	20,644,227.63
41	9-(3-Methyl-5-pentyl-2-furyl)nonanoic acid	C_19_H_32_O_3_	0.53	308.2353	25.100	20,430,640.33
42	Capsiamide	C_17_H_35_NO	0.46	269.2720	29.675	19,317,050.54
43	Rolipram	C_16_H_21_NO_3_	1.46	275.1525	19.262	18,929,272.68
44	(2E,4E,16Z)-1-(1-Piperidinyl)-2,4,16-icosatrien-1-one	C_25_H_43_NO	−0.34	373.3343	30.555	18,364,462.94
45	Myxopyronin B	C_24_H_33_NO_6_	1.00	431.2312	18.027	17,849,057.48
46	γ-Butyrobetaine	C_7_H_15_NO_2_	0.83	145.1104	1.138	17,329,421.76
47	2,12-Bis[(dimethylamino)methyl] cyclododecanone	C_18_H_36_N_2_O	0.30	296.2829	28.372	16,890,673.47
48	Tranexamic acid	C_8_H_15_NO_2_	1.16	157.1105	1.173	16,871,292.77
49	1-linoleoyl-sn-glycerol-3-phosphoethanolamine	C_23_H_44_NO_7_P	1.09	477.2861	18.363	15,973,939.02
50	Octinoxate	C_18_H_26_O_3_	−0.13	290.1882	19.277	15,663,180.24
51	Nicotinuric acid	C_8_H_8_N_2_O_3_	0.63	180.0536	1.528	15,451,169.22
52	(2-{2-[5-(4-Tert-butyl-cyclohexyl oxy)-2,3-dihydro-indol-1-yl]-2-oxo-ethylamino}-ethyl)-phosphonicacid diethyl ester	C_26_H_43_N_2_OP	3.73	494.2928	29.343	15,244,360.56
53	Guanidine carbamate	C_2_H_8_N_4_O_2_	1.50	120.0649	1.102	14,925,123.37
54	Piracetam	C_6_H_10_N_2_O_2_	1.13	142.0744	1.134	14,436,868.86
55	Terodiline	C_20_H_27_N	0.62	281.2145	26.393	14,000,431.15
56	N-Benzyloleamide	C_25_H_41_NO	0.79	371.3191	28.304	13,479,113.87
57	L-gamma-Glutamyl-L-valine	C_10_H_18_N_2_O_5_	0.42	246.1217	2.133	13,380,919.38
58	Threo-Sphingosine, (−)-	C_18_H_37_NO_2_	0.81	299.2827	17.925	13,264,548.93
59	N-Methyl tetradecanamide	C_15_H_31_NO	0.98	241.2408	27.170	12,944,705.58
60	8-decyl octadecyl N-[4-(dimethyl amino)butyl]-N-methylcarbamate	C_36_H_74_N_2_O_2_	0.16	566.5751	28.150	12,118,497.59
61	Zalcitabine	C_9_H_13_N_3_O_3_	1.37	211.0960	1.157	5,421,160.08

Note: Area sample represents the relative peak-area percentage of the identified compounds.

**Table 2:** Analysis of 16 bioactive compounds identified in *Lissachatina fulica* mucin (LFM) and their predicted antiatopic dermatitis–related targets.

	Bioactive compound	Target gene/protein	Target class	Biological process (gene ontology) enrichment
1	Macamide	CCL2—C-C motif chemokine ligand 2	Family A G protein-coupled receptor	Chemokine-mediated signaling pathway; Leukocyte, neutrophil, lymphocyte, monocyte, eosinophil, and dendritic cell chemotaxis; Mononuclear cell and myeloid leukocyte migration; Cellular response to interleukin-1
2	Eplerenone	TNF—Tumor necrosis factor	Secreted protein	Tumor necrosis factor-mediated signaling pathway; Cytokine-mediated signaling pathway; Positive regulation of I-κB kinase/NF-κB signaling; Apoptotic signaling pathway
		PTGS2—Prostaglandin-endoperoxide synthase 2 (COX-2)	Oxidoreductase	Unsaturated fatty acid biosynthetic process; Fatty acid, prostaglandin, and eicosanoid biosynthetic process; Cyclooxygenase pathway; Regulation of acute inflammatory response
3	Artocarpin	PTGS2—Prostaglandin-endoperoxide synthase 2 (COX-2)	Oxidoreductase	Same target as above
4	1,4-dimethylcyclohexane;1-(3-methylpentyl)-4-phenoxybenzene; N,N,2-trimethyl-5H-1,3-benzodiazepin-4-amine	PTGS2—Prostaglandin-endoperoxide synthase 2 (COX-2)	Oxidoreductase	Same target as above
5	2a,7-Dihydroxy-3-(hydroxymethyl)-6,6,7b-trimethyl-2,2a,4a,5,6,7,7a,7b-octahydro-1H-cyclobuta[e]inden-2-yl 3-chloro-4,6-dihydroxy-2-methylbenzoate	MMP9—Matrix metallopeptidase 9	Protease	Negative regulation of metallopeptidase activity; Cellular response to UV-A; Negative regulation of endopeptidase activity; Extracellular matrix organization; Response to cytokine; Regulation of apoptotic signaling pathway
6	1-stearoyl-2-hydroxy-sn-glycero-3-phosphoethanolamine	AKT1—AKT serine/threonine kinase 1	Kinase	Cellular response to oxygen levels; Response to osmotic stress; Regulation of protein kinase B signaling; Response to temperature stimulus; Response to heat; Cellular response to peptide; Response to insulin
7	2-(3-Phenylpropyl)pyridine	PTGS2—Prostaglandin-endoperoxide synthase 2 (COX-2)	Oxidoreductase	Same target as above
8	1-Palmitoyl-sn-glycero-3-phosphocholine	AKT1—AKT serine/threonine kinase 1	Kinase	Same target as above
9	1-palmitoyl-2-hydroxy-sn-glycero-3-phosphoethanolamine	AKT1—AKT serine/threonine kinase 1	Kinase	Same target as above
10	Spisulosine	AKT1—AKT serine/threonine kinase 1	Kinase	Same target as above
11	Capsiamide	PTGS2—Prostaglandin-endoperoxide synthase 2 (COX-2)	Oxidoreductase	Same target as above
12	Rolipram	ICAM1—Intercellular adhesion molecule-1	Adhesion	Leukocyte cell-cell adhesion; Membrane to membrane docking; Cell adhesion mediated by integrin; Cellular extravasation; Cell-cell adhesion via plasma-membrane adhesion molecules; Cell-cell adhesion; Cell-matrix adhesion; Leukocyte activation
13	Myxopyronin B	SYK—Spleen tyrosine kinase	Kinase	Stimulatory C-type lectin receptor signaling pathway; Immune response-activating cell surface receptor signaling pathway; Positive regulation of immune response; Fc-gamma receptor signaling pathway involved in phagocytosis; Positive regulation of innate immune response; Myeloid cell activation involved in immune response
14	Octinoxate	AKT1—AKT serine/threonine kinase 1	Kinase	Same target as above
15	(2-{2-[5-(4-Tert-butyl-cyclohexyloxy)-2,3-dihydro-indol-1-yl]-2-oxo-ethylamino}-ethyl)-phosphonic acid diethyl ester	AKT1—AKT serine/threonine kinase 1	Kinase	Same target as above
		SYK—Spleen tyrosine kinase	Kinase	
16	N-Benzyloleamide	CCL2—C-C motif chemokine ligand 2	Family A G protein-coupled receptor	Same target as above
